# Draft Genome Sequences of Five Clinical *Enterococcus cecorum* Strains Isolated from Different Poultry Species in Poland

**DOI:** 10.1128/genomeA.01082-15

**Published:** 2015-09-17

**Authors:** Beata Dolka, Rikke Heidemann Olsen, Ida Cecilie Naundrup Thøfner, Jens Peter Christensen

**Affiliations:** a Department of Pathology and Veterinary Diagnostics, Faculty of Veterinary Medicine, Warsaw University of Life Sciences – SGGW, Warsaw, Poland; b Department of Veterinary Disease Biology, Faculty of Health and Medical Sciences, University of Copenhagen, Copenhagen, Frederiksberg C, Denmark

## Abstract

Here, we report five draft genome sequences of *Enterococcus cecorum* strains that were isolated from different bird species of affected poultry flocks (commercial broilers [CB], broiler breeders [BB], commercial layers [CL], ducks [D], and geese [G]) in Poland.

## GENOME ANNOUNCEMENT

*Enterococcus cecorum* is known as a member of the commensal intestinal microbiota in healthy humans and animals, but it can act as a pathogen. From 2002 until today, a growing number of *E. cecorum*-associated disease outbreaks in poultry flocks, especially broilers and broiler breeders, have been reported worldwide. The most predominant lesions are enterococcal spondylitis (ES), osteomyelitis, arthritis, femur head necrosis (FHN), and bacteremia (1–5). Recently, genomic analysis revealed fundamental differences in pathogenic isolates from chickens in the southeastern United States (6).

In the present study, *E. cecorum* strains were cultivated from tissue samples taken from affected poultry. Bacteria were identified as *Enterococcus* based on their phenotypic properties. Identification to the species level was done using API rapid ID 32 Strep (bioMérieux, France), PCR amplification of the *sodA* (superoxide dismutase) fragment gene, and sequencing of PCR products (7). BLAST search results showed 99 to 100% sequence identity with the existing GenBank *E. cecorum* sequences. We found no high-level gentamicin resistance (HLGR) or vancomycin*-*resistant enterococci (VRE) in enterococcal strains after using the standard disk diffusion method. *E. cecorum* strain BB-66 exhibited intermediate susceptibility to vancomycin.

Here, we present five draft genome sequences of *E. cecorum* clinical strains from different poultry species in Poland: commercial broilers (CB-32), broiler breeders (BB-66), commercial layers (CL-1), ducks (D-104), geese (G-29).

Good-quality genomic DNA was isolated from pure cultures of *E. cecorum* CB-32, BB-66, CL-1, G-29, and D-104 and submitted for sequencing. Five genomes were sequenced by the Illumina paired-end method (MiSeq) using a paired-end library (TruSeq Nano DNA), with an average read length of 2 × 150 bp. The reads were trimmed for quality and *de novo* assembled using CLC Genomics Workbench 7.0.

The results of the sequencing are summarized in Table 1. The genome sequences were annotated by the Rapid Annotations using Subsystems Technology (RAST) server (8) and by the NCBI Prokaryotic Genome Annotation Pipeline (PGAP) (9).

**TABLE 1:** Summary of genome sequencing in the present study

*E. cecorum* isolate	Reads (Mb)	Fold coverage	No. of scaffolds	Genome size (bp)	G+C (%)	Accession no.
CB-32	132.6	57.8	61	2,293,164	36.5	LDEB00000000
BB-66	162.2	65.4	62	2,480,260	36.2	LDED00000000
CL-1	143.8	61.5	47	2,337,819	36.3	LDEA00000000
G-29	171.9	80.5	46	2,133,362	36.9	LDEC00000000
D-104	149.9	65.8	28	2,275,377	36.3	LDDZ00000000

The CB-32 genome consists of 2,267 genes (51 tRNAs and 3 rRNAs) and 2,113 expected coding sequences (CDSs). The genome of BB-66 consists of 2,479 genes (47 tRNAs and 3 rRNAs), of which 2,310 are coding sequences (CDSs). For CL-1, we identified 2,315 genomic features consisting of 2,163 coding sequences (predicted), 40 tRNAs, and 6 rRNAs. The D-104 genome consists of 2,269 genes (55 tRNAs and 10 rRNAs) and 2,115 CDSs. For the G-29 genome, we revealed 2,158 genes (46 tRNAs and 5rRNAs), including 1,987 CDSs.

To the best of our knowledge, this is the first genome report of clinical *E. cecorum* strains isolated from different poultry species in Europe. The sequence data presented here will contribute to understanding the pathogenicity of *E. cecorum* against poultry.

### Nucleotide sequence accession numbers.

The five whole-genome shotgun projects have been deposited at DDBJ/EMBL/GenBank under the accession numbers listed in Table 1. The versions described in this paper are the first versions.
